# Can Product Information Steer towards Sustainable and Healthy Food Choices? A Pilot Study in an Online Supermarket

**DOI:** 10.3390/ijerph19031107

**Published:** 2022-01-19

**Authors:** Nadine E. van der Waal, Frans Folkvord, Rachid Azrout, Corine S. Meppelink

**Affiliations:** 1 Department of Communication and Cognition, Tilburg School of Humanities and Digital Sciences, Tilburg University, 5037 AB Tilburg, The Netherlands; f.folkvord@tilburguniversity.edu; 2 Open Evidence Research, 08005 Barcelona, Spain; 3 Amsterdam School of Communication Research (ASCoR), University of Amsterdam, 1012 WX Amsterdam, The Netherlands; r.azrout@uva.nl (R.A.); c.s.meppelink@uva.nl (C.S.M.)

**Keywords:** sustainable purchase behavior, perceived consumer effectiveness, green skepticism, product information, sustainability claims, health claims

## Abstract

Sustainable dietary choices have become increasingly important because of the current environmental threats the world is facing. Nonetheless, consumers find it difficult to assess a product’s sustainability and therefore make better choices. This pilot study tested whether explanatory product information about sustainability increased sustainable purchases in an online supermarket and whether additional health information increased message effectiveness. Perceived consumer effectiveness (i.e., the perception of the degree to which individual actions can contribute to environmental problems) and green skepticism were hypothesized to mediate the effect of message type, and environmental attitudes were included as the moderator. An experiment using a one-factor design was conducted among 101 participants who were assigned to one of three experimental conditions: sustainability claim only, explanatory sustainability claim, and explanatory sustainability and health claim. Analyses showed that an explanatory sustainability claim (regardless of whether this claim was accompanied by a health claim) led to fewer sustainable purchases through perceived consumer effectiveness but only for those with low environmental attitudes. No effects were found for the addition of a health claim. The results from this pilot provide insight for future studies that aim to examine how online supermarkets should communicate to increase sustainable purchases.

## 1. Introduction

During the last few decades, it has become apparent that there is a pressing need to make a global change towards healthier and more sustainable lifestyles [1,2]. More specifically, our current food systems has great direct impact on the environment, accounting for 25% of all CO_2_ emissions worldwide [3]. As a consequence, the Dutch National Institute for Public Health and the Environment recently sounded the alarm that Dutch citizens need to face the challenge of creating a more sustainable consumption pattern [3]. This is also acknowledged by leading experts worldwide [1].

People’s environmental concerns are substantial, according to a Eurobarometer survey conducted in 2021: 93% of citizens in all EU member states agreed that climate change is a serious problem in the EU [4]. Furthermore, almost one third appeared to be willing to adjust dietary habits, in case this contributes against climate change [4]. Despite consumers’ positive attitudes and their willingness to change their consumption patterns, people do not engage often in actual sustainable purchase behavior; this phenomenon is known as the attitude-behavior gap (e.g., [5,6,7]). This pilot study aims to explore the conceptual idea that providing point-of-purchase product information could foster sustainable purchases, by arguing that clear explanations about the sustainability of a product will ensure more guidance for consumers to make informed decisions and to act in line with their attitudes.

The ambiguous definition of sustainable consumption and the uncertainty of what it entails from a consumer’s perspective could be factors that enlarge the attitude-behavior gap [8]. Assessing sustainable attributes of a product seems hard for consumers, as the benefits of sustainable products are often poorly communicated by governments and companies [7]. Different studies have pointed out the importance of providing accurate, simplistic, and sufficient information regarding sustainable products, such as package labelling, so that consumers can make well-considered purchase decisions [7,9]. The wide variety of different sustainability labels potentially gives rise to issues of reliability, information overload, confusion, or incoherence [10]. For example, a quasi-experimental study showed that eco-label confusion leads to distrust in, and dissatisfaction with, the product [11]. In this pilot study, it is therefore argued that providing clear explanatory product information about the sustainability of a product could increase sustainable purchases, such as explicit mention of the reason why a product is considered sustainable. In addition to providing information about sustainability attributes, previous research suggests that communicating health attributes might be an efficient strategy for increasing sustainable consumption [12,13]. Specifically, perceptions of a food product based on its front-of-package information, such as perceptions of the quality of food products or healthiness of a food product, are considered to be important factors for choosing a sustainable product [10].

Sustainable purchase behavior is typically something that is in the collective interest but comes with individual sacrifices, such as a higher product price, increased effort searching for a product, or spending more time to evaluate a product [14]. Social dilemma theory [15] states that consumers often base their decisions on short-term goals and individual interests because acting in the interest of collective interest often accompanies a sacrifice in the short term. However, if all individuals act on the collective interest, every individual will eventually benefit from this in the long run (e.g., the preservation of the Earth’s resources). Because the success of the collective efforts depends on whether all individuals engage in these efforts, a (social) dilemma arises. This dilemma could potentially be solved by using health attributes, in addition to sustainability attributes, so that individual interests could become more prevalent, and individuals could therefore have more reason to purchase sustainable products.

This study examines the effects of an explanatory sustainability and health claim, an explanatory sustainability claim, and a sustainability claim only. To clarify this, in the sustainability claim only conditions, products are marked as “sustainable choice”, whereas in the explanatory conditions, the exact explanation is given for why a product is sustainable (and healthy). It is expected that a combination of an explanatory sustainability and a health claim leads to more sustainable purchases than an explanatory sustainability claim and a sustainability claim only (H1a). Furthermore, it is expected that an explanatory sustainability claim leads to more sustainable purchases than a sustainability claim only (H1b).

Next, the literature shows that perceived consumer effectiveness (PCE), which is defined as the perception of the degree to which individual actions can contribute to environmental problems [16], is a determinant for sustainable purchase intention and sustainable purchases [17,18]. Several studies highlighted that consumers typically report that their consumption behavior has a limited impact on the environment [10,19]. This study argues that communicating the environmental benefits of a product can contribute to an increase in PCE, as people become aware that buying the product leads to positive outcomes [7,14]. Therefore, it is expected that an explanatory sustainability claim leads to higher levels of PCE than a sustainability claim only (H2a) and that this is positively related to sustainable purchase behavior (H2b).

In addition to lacking PCE, which inhibits consumers purchasing of sustainable products, consumers’ skepticism towards green claims is another possible explanation [20,21]. Green skepticism is defined as a distrust towards green claims made by firms [21], which is partly due to incidents of greenwashing. Companies sometimes falsely portray their products or services as environmentally friendly, with an aim to increase profit instead of to support the environment [22]. Leonidou and Skarmeas [20] recommend firms disclose all the information necessary to support the performance of their sustainable products, since skeptical people are characterized by their ability to quickly change their minds when confronted with compelling evidence [21]. For example, skeptical consumers have been found to actively seek additional information on the sustainability of products, e.g., by reading certifications, asking friends, and accessing websites and discussion groups [20]. These effects might also occur in the opposite direction, such that providing consumers with explanatory product information could reduce consumers’ green skepticism. It is therefore expected that an explanatory sustainability claim leads to lower levels of green skepticism than a sustainability claim only (H3a) and that an explanatory sustainability claim increases sustainable purchase behavior by reducing green skepticism, whereas a sustainability claim only does not increase sustainable purchases through green skepticism (H3b).

Matthes and Wonneberger [23] argue that skepticism can be considered a trait whereby a distinction can be made between green versus non-green consumers. Green consumers appear to accept individual responsibility and accountability for the provision of public goods more than non-green consumers [24]. In addition, information utility has been shown to be an important variable in explaining the effects of green skepticism [25]. Namely, green consumerism was positively associated with information utility and negatively associated with green skepticism [23]. Thus, green consumers value the information in a claim as more useful, therefore experiencing less skepticism towards the environmental claims. In line with this, it is assumed that the effect of an explanatory sustainability claim, compared to a sustainability claim, is stronger for consumers with high environmental attitudes compared to those with low environmental attitudes (H3c).

## 2. Materials and Methods

### 2.1. Design

A pilot using a one-factor experimental design was conducted among 101 participants who were assigned to one of the three experimental conditions (sustainability claim only, *n* = 33; explanatory sustainability claim without health claim, *n* = 35; and explanatory sustainability claim with health claim, *n* = 33). Data were collected over a four-week period. Randomization checks were performed including the variables age, gender, and education level, whereby no differences in these variables between the three experimental conditions were found. A MANOVA showed that participants were equally distributed across the three conditions when considering age (*F* (2, 98) = 2.99, *p* = 0.055), gender (*F* (2, 98) = 1.07, *p* = 0.348), and educational level (*F* (2, 98) = 0.08, *p* = 0.921).

### 2.2. Participants and Recruitment

Participants were recruited at the campus of a large Dutch university through flyers via the university’s human subject pool and the researchers’ personal networks. Only Dutch-speaking people could participate in the study. The final sample was aged 18–56 years (*M* = 25.57, *SD* = 9.60), and the majority of the sample was female (77.2%). Most participants were enrolled in a bachelor’s study (53.5%) or had finished a bachelor’s (22.8%) or a master’s degree (12.9%). The study design was approved by the Ethics Committee of the Amsterdam School of Communication Research (reference number 2019-PC-20432, 8 April 2019). Communication Science students were rewarded with course credits for their contribution (see Table 1 for sample descriptives per condition).

### 2.3. Stimulus Material and Manipulation

A simplified, simulated version of the largest Dutch online supermarket was created with the website builder tool www.jouwweb.nl. The logo, products, and prices were similar to those of the actual Dutch supermarket. The online supermarket included 33 products distributed over 6 product categories (see Table 2). The product categories, including bread, dairy and sandwich filling, pasta, vegetables, meat and vegetarian alternatives, and sauces, were selected based on the shopping task which participants had to fulfil. Other than the product information, the online supermarket was held constant across the conditions. At least one product per product category contained product information, with a total of ten products (see Table 2).

Three different versions of the online supermarket were created. In the sustainability claim only condition, participants were exposed to a sustainability claim (i.e., “sustainable choice”). In the explanatory sustainability claim without health claim condition, the product information included a brief explanation of why the product would be a sustainable choice (e.g., “Seasonal veggie! This spinach is produced in the Netherlands, so no unnecessary CO_2_ emissions due to transportation.”). In the explanatory sustainability with health claim condition, the sustainable attribute of the former condition was supplemented with a health attribute of the product (e.g., “Spinach is rich in iron and promotes the immune system.”). The product information was designed according to information provided by The Netherlands Nutrition Centre (e.g., [26]). The content of the website was modified daily to one of the three conditions.

### 2.4. Manipulation Checks

The first manipulation check focused on participants’ recollection of the product information. A chi-square test revealed that the participants correctly recalled the product information. In the sustainability and health condition, participants significantly more often answered that the product information focused on sustainability and health, whereas in the sustainability conditions, participants indeed answered that the product information focused on sustainability, *χ*^2^ (4, *N* = 101) = 15.08, *p* = 0.005. The relationship was moderately strong, Cramers *V* = 0.27.

To ensure that perceived message length would not be a confounder in our study, participants were asked to assess the quantity of the information on a seven-point Likert scale. Results from a one-way ANOVA showed that participants did not perceive any differences in information quantity between the sustainability claim only condition (*M* = 3.03, *SD* = 0.85), the explanatory sustainability claim condition (*M* = 2.83, *SD* = 0.66), and the explanatory sustainability and health claim condition (*M* = 3.03, *SD* = 0.59), *F* (2, 98) = 0.93, *p* = 0.397. Hence, it can be concluded that there were no differences in participants’ perceptions of information quantity across the different conditions.

Lastly, the credibility of the product information was assessed on a five-point semantic differentiation scale with four items (e.g., true/false). A one-way ANOVA revealed no significant differences between the sustainability claim only condition (*M* = 4.58, *SD* = 0.59), the explanatory sustainability claim condition (*M* = 4.65, *SD* = 0.70), and the explanatory sustainability and health claim condition (*M* = 4.61, *SD* = 0.61), *F* (2, 98) = 0.12, *p* = 0.891. Thus, participants perceived the product information to be equally reliable across the three conditions.

### 2.5. Pre-Test

A pre-test was conducted among 34 respondents, during which they were exposed to eleven products with product information. Each product was provided with either a sustainability claim only, an explanatory sustainability claim without health claim, or an explanatory sustainability claim with health claim. For each product, respondents had to indicate what the product information was about, by answering a multiple-choice question with several topics as answer categories. Next to that, respondents had to indicate on a seven-point Likert scale whether they found the information trustworthy. A chi-square test was performed to examine the relationship between information topic and the topic that participants associated with the information. Indeed, respondents answered that the topic was about sustainability and health more often in the explanatory sustainability claim with health claim condition. Furthermore, respondents indicated that the information was about sustainability in the sustainability claim only and the sustainability claim with information conditions, *χ*^2^ (4) = 121.82, *p* < 0.001. The relationship appeared to be strong, Cramer’s *V* = 0.55. Furthermore, a paired sample *t*-test revealed that respondents perceived no significant difference in reliability between the products with sustainable information (*M* = 3.75, *SD* = 0.65) and products with sustainable and health information (*M* = 3.67, *SD* = 0.66), *t* (304) = −1.00, *p* = 0.316.

### 2.6. Procedure

Participants were welcomed in a quiet room where they first provided their informed consent and were assured of participant anonymity, the possibility to withdraw their permission to allow their data to be used, as well as the guarantee that they would not be subjected to any risk or discomfort. After agreeing to participate, demographics, control variables, and environmental attitudes were assessed. Then, participants read written instructions before they started their grocery shopping online. People were instructed to buy ingredients for a four-person pasta meal, including at least one pasta product, one kind of vegetable, and one type of sauce. After completion of the task, they returned to the questionnaire, where they answered questions about PCE, green skepticism, behavioral intentions, and the manipulation check. Finally, participants were debriefed by disclosing the actual goal of the study and by explaining that the online supermarket was designed for this study.

### 2.7. Measures

In Appendix A (Table A1), all items included in the questionnaire are provided.

#### 2.7.1. Dependent Variable: Sustainable Purchase Behavior

Sustainable purchase behavior was measured as a sum score of the chosen products with product information. The number of products that participants chose ranged from zero to six products (*M* = 2.27, *SD* = 1.46).

#### 2.7.2. Mediator: Perceived Consumer Effectiveness

To measure PCE, the original four-item scale from Roberts [16] was used, containing items such as “It is worthless for the individual consumer to do anything about pollution” and “When I buy products, I try to consider how my use of them will affect the environment and other consumers”. Two items were reverse coded, and all items were measured on a seven-point Likert scale. Due to low factor loading of the item ‘Whenever I buy products, I try to consider what the purchase does to the environment and to other people’ (b* = 0.36), this item was excluded from further analyses. The descriptives reported reflect the values of the final scale. The final scale consisted of three items (*M* = 5.42, *SD* = 1.03, α = 0.69).

#### 2.7.3. Mediator: Green Skepticism

Green skepticism was measured by a scale adopted from Fenko, Kersten, and Bialkova [27], which was originally used to measure green advertising and label skepticism. The items were slightly reformulated, and two items were removed to fit the current study about product information. Participants were explicitly told that the questions referred to product information that was provided in the online supermarket. Examples of items are “Green claims are intended to mislead rather than to inform consumers” and “Green claims do not tell much useful information about products”. Eight items were measured on a seven-point Likert scale, of which four were reverse coded. The item ‘The green claims do not tell much useful information about products’ (b* = 0.36) was removed due to low factor loading. Seven items were included in the final scale (*M* = 3.31, *SD* = 1.01, *α* = 0.90).

#### 2.7.4. Moderator: Environmental Attitudes

The twelve items to measure attitudes about environmental issues were derived from a study of Kilbourne, Beckmann, and Thelen [28]. The items measured attitudes towards general environmental problems (e.g., “Global warming is not really a problem”), shortages (e.g., “Agricultural productivity will decline in the near future”), and extinctions (e.g., “Destruction of rainforests will have long term environmental consequences”) on a seven-point Likert scale. Solely the scale measuring environmental problems were included in the analyses, since the subscales shortages (*α* = 0.60) and extinctions (*α* = 0.58) were not sufficiently reliable. Furthermore, due to low factor loading of the item ‘In a crisis, industries will develop solutions to environmental problems’ (b* = 0.37), this item was excluded from further analyses. The final subscale measuring attitudes towards environmental problems consisted of four reverse-coded items and scores ranging from 3.50 to 7.00 (*M* = 5.70 *SD* = 0.93, *α* = 0.67). A high score on this scale is referred to as ‘high environmental attitudes’, whereas a low score is referred to as ‘low environmental attitudes’ in this paper. This indicates that those scoring higher on the scale acknowledge the environmental problems to a stronger degree than those scoring lower on the scale.

As the hypothesis distinguishes between individuals with high and low environmental attitudes, the subscale was recoded into two groups using the median split method (low vs. high environmental attitudes, median = 5.75).

### 2.8. Analytical Strategy

To test H1a and H1b, an ANOVA is conducted with sustainable purchases as the dependent variable and the experimental variable with three conditions as a predictor. H2a through H3c are tested with structural equation modelling, since this analysis method is useful when assessing models that include multiple indirect effects.

To test hypotheses H2a through H3c, the explanatory claim without health claim condition and the explanatory claim with health claim condition are merged into one explanatory condition. This method is employed because the aim of these mediations is to test whether it is the presence or absence of an explanation that affects PCE and green skepticism (regardless of whether this includes, or does not include, health information). Merging the two conditions would lead to more power to detect significant effects.

**Figure 1:**
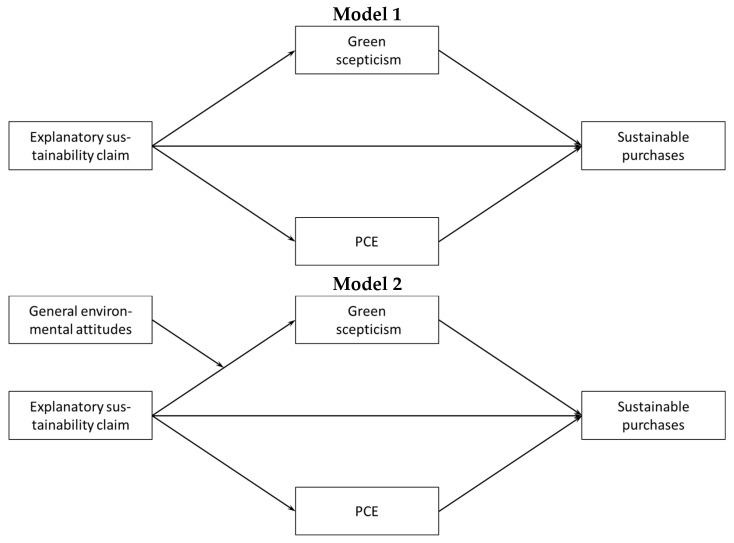
Baseline path models. Note: In these models, the two explanatory sustainability claims (with and without the health claim condition) are merged. In Model 1, general environmental attitudes are added as a control variable.

In Figure 1, the models that are used to test H2a through H3b (Model 1: upper panel) and H3c (Model 2: lower panel) are depicted. Model 2 included an interaction term of the experimental variable and general environmental attitudes. This interaction term was added as a predictor of green skepticism. Correlations between the variables are provided in Table 3.

### 2.9. Assessing Model Fit

Before model construction, data were inspected, and several assumptions were checked for. Variables included in the model were normally distributed with skewness between −1 and +1 and kurtosis between −3 and +3. The dataset did not contain ill-scaled variances. Variables were linearly associated, and there were no signs of multicollinearity or extreme outliers. It should be noted that the amount of cases preferably should be equal to twenty per parameter (Kline, 2011) and that this assumption could not be met due to practical constraints.

**Figure 2:**
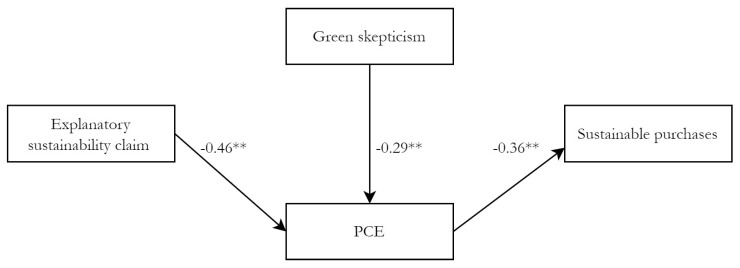
Final path model to test H1b through H3b (Model 1). Note: ** Relationship is significant at the 0.01 level. Unstandardized coefficients are reported. General environmental attitude is included as control variable but not shown for reasons of clarity. Model fit: *χ*^2df=4^ = 4.64, *p* = 0.327, CFI = 0.98, RMSEA = 0.04, 90%CI = [0.00, 0.16], PCLOSE = 0.451.

**Figure 3:**
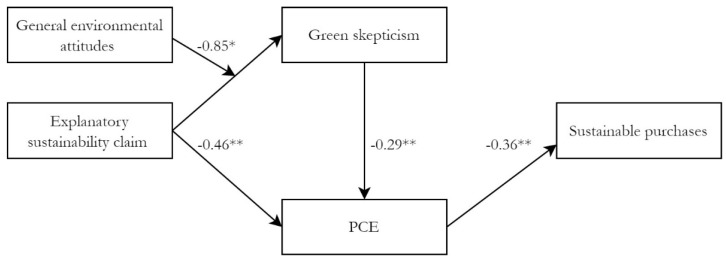
Final path model to test H3c (Model 2). Note: ** Relationship is significant at the 0.01 level. * Relationship is significant at the 0.05 level. Unstandardized coefficients are reported. Model fit: *χ*^2df=4^ = 2.89, *p* = 0.576, CFI = 1.00; RMSEA = 0.00, 90%CI = [0.00, 0.13], PCLOSE = 0.684.

As a first step, model fit was assessed. Good fit is established when the model chi-square is not significant, the comparative fit index (CFI) is above 0.95, the root mean square error of approximation (RMSEA) is below 0.06 with the upper-bound of the 90% confidence interval below 0.10, and the *p*-value of close fit (PCLOSE) is not significant [29,30]. Using the fit indices, it is decided which paths in the model are necessary for model fit, thus establishing which direct and indirect effects are relevant. Apart from this, indirect effects are also evaluated with another test, based on 5000 bootstraps using the bias-corrected percentile method. Table 3 provides the correlations between the variables. Model 1 initially had insufficient fit, which was caused by the high residual correlation between green skepticism and PCE. The addition of a path between green skepticism and PCE would be an evidence-based choice, considering a previous study that has reported a correlation between green skepticism and PCE [23]. Albayrak and colleagues [31] also investigated the relationship between green skepticism and PCE and found that green skepticism had a negative effect on PCE, which in turn affected green purchase behavior. Hence, a path is added from green skepticism to PCE, which creates a just-identified model with perfect fit. Several paths could be removed without causing problems or significant loss of model: the path from general environmental attitudes to green skepticism, the path from green skepticism to sustainable purchases, and the paths from the experimental conditions to green skepticism and sustainable purchases. The final model, with a good fit, is presented in Figure 2, and the fit indices for each re-specification step can be found in Table 4. For Model 2, the fit was also insufficient due to the absence of a path from green skepticism to PCE. Adding this path significantly improved model fit (*χ*^2^_diff-df=1_ = 8.84, *p* = 0.003). Again, several paths could be removed without significant loss of model fit: the direct effect of the experimental variable on sustainable purchases and the path from green skepticism to sustainable purchases (*χ*^2^_diff-df=2_ = 1.74, *p* = 0.418). The final model, which is shown in Figure 3, has a good fit.

## 3. Results

In H1a and H1b, it was hypothesized that an explanatory sustainability with health claim would lead to more sustainable purchases than an explanatory sustainability claim without health claim and that an explanatory sustainability claim without health claim would lead to more sustainable purchases than a sustainability claim only. To test this hypothesis, an ANOVA was conducted, which revealed no significant differences in sustainable purchases between the three conditions, *F* (2, 98) = 2.28, *p* = 0.108, *η*^2^ = 0.04. This indicates that the explanatory sustainability claim with (*M* = 2.00, *SD* = 1.48) and without health claim conditions (*M* = 2.69, *SD* = 1.41) do not result in significantly more sustainable purchases than the sustainability claim only condition (*M* = 2.09, *SD* = 1.44). Therefore, H1a and H1b are rejected.

To analyze H2a, which predicted that an explanatory sustainability claim would lead to higher levels of PCE than a sustainability claim only, Model 1 was used. The model indicates a significant effect of an explanatory sustainability claim on PCE (*b* = −0.46, *SE* = 0.20, *p* = 0.021). This coefficient relates to the differences between the model-implied means of the two conditions: respondents in the explanatory sustainability conditions (*M* = 5.27) score lower on PCE than respondents in the claim only condition (*M* = 5.72). This clearly is at odds with H2a, which predicted a lower PCE in the claim only condition, compared to the explanatory conditions.

H2b predicted a mediation effect, in which it was expected that increased levels of PCE would lead to more sustainable purchases. The only path from explanatory sustainability claims to sustainable purchases ran through PCE in the model, and, indeed, this indirect effect equals the total effect: *b* = −0.16, *SE* = 0.10, *p* = 0.022, 95%CI = [−0.43, −0.02]. This positive effect of PCE on sustainable purchases confirms H2b. PCE functions as a full mediator in this model, although not in the expected direction.

In H3a, it was expected that an explanatory sustainability claim would lead to lower levels of green skepticism than a sustainability claim only. No direct or indirect effects of an explanatory sustainability claim on green skepticism were necessary for model fit, which implies that no support is found for H3a. Thereby, H3b is also rejected, in which it was predicted that green skepticism would function as a mediator between claim type and sustainable purchases. However, due to re-specification, an indirect effect of green skepticism on sustainable purchases was found through PCE: *b* = −0.10, *SE* = 0.06, *p* = 0.015, 95%CI = [−0.25, −0.02].

H3c expected that the relationship between information type and green skepticism was moderated by environmental attitudes, which was tested with Model 2. The effect of the interaction term is significant (*b* = −0.85, *SE* = 0.42, *p* = 0.041), implying that the effect of explanatory sustainability claims on green skepticism is moderated by general environmental attitudes. The direction of the coefficient is also in the expected direction; for individuals with high general environmental attitudes, explaining the claim leads to less skepticism compared to individuals with low general environmental attitudes. To further scrutinize this moderation, the marginal effects per value of the moderator are outlined.

**Figure 4:**
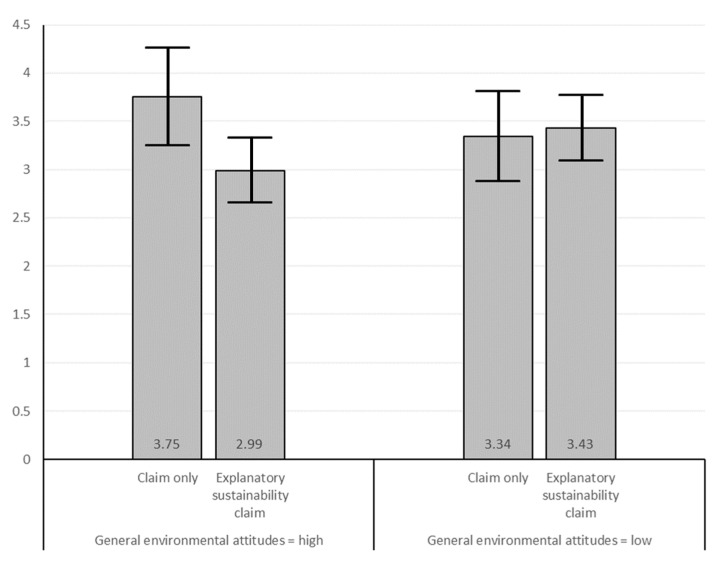
Model implied means of green skepticism with 95% confidence intervals.

As Figure 4 shows, among individuals with high general environment attitudes, the mean of green skepticism is lower in the explanatory sustainability claims condition compared to the sustainability claim only condition, and this difference is significant: *b* = −0.76, *SE* = 0.30, *p* = 0.012. This indicates that consumers who hold high environmental attitudes are becoming less skeptical when more explanatory information is provided, compared to when only a sustainability claim is made. Among individuals with low general environmental attitudes, the mean of green skepticism is higher in the explanatory sustainability claim conditions compared to the sustainability claim only condition, but this difference is not significant (*b* = 0.09, *SE* = 0.28, *p* = 0.759). This implies that consumers with low environmental attitudes are hardly affected in their level of skepticism through an explanation of the sustainability claim, while those with high general environmental attitudes become less skeptical. This is in accordance with the expectation in H3c. We discuss the implication of this finding in the discussion.

The significant interaction with significant marginal effects on green skepticism, in combination with the added effect from green skepticism to PCE, has some important implications for the total effects of the stimuli on both PCE and sustainable purchases. Given that the total effects are partly indirect through green skepticism and that this effect is moderated, the total effects are also likely to be moderated. Looking at these moderated total effects, we find that among individuals with low general environmental attitudes the explanatory sustainability claim conditions have more negative total effects on PCE (*b* = −0.48, *SE* = 0.23, *p* = 0.036, 95%CI = [−0.92, −0.03]) than among individuals with high environmental attitudes (*b* = −0.24, *SE* = 0.21, *p* = 0.233, 95%CI = [−0.65, 0.16]). The same can be found for sustainable purchases (low general environmental attitudes: *b* = −0.17, *SE* = 0.11, *p* = 0.025, 95%CI = [−0.46, −0.19]: high general environmental attitudes: *b* = −0.24, *SE* = 0.21, *p* = 0.233, 95%CI = [−0.65, 0.16]). Furthermore, both the total effect on PCE and sustainable purchases are significant among those with low general environmental attitudes and not significant among those with high general environmental attitudes. These results suggest that among those with low general environmental attitudes, the addition of explanatory information does have a significant impact on their sustainable purchasing behavior.

### Alternative Models

The conclusion that the addition of explanatory information among those with low environmental attitudes has a significant impact on their sustainable purchasing behavior weighs heavily on the re-specifications made to the model, i.e., adding the path from green skepticism to PCE. Although the direction of this path seems logical, the evidence is not overwhelming [31]. In contrast, previous research has suggested a relationship from PCE to green skepticism [23].

Therefore, models were tested in which the high residual correlations were solved in the initial model by adding a path in the opposite direction (see Table 5 for the fit indices). As these models are not nested, comparison of the model is only possible by eyeballing the differences between the fit indices. For the main effects model, the model with a path from PCE to green skepticism has slightly higher values of the chi-square, RMSEA, and Akaike’s information criterion, as well as a slightly lower value of the CFI. This might imply that the model presented above, with a path from green skepticism to PCE, has a better fit. However, the differences are very minimal, and except for the RMSEA, all fit indices of the alternative model reach levels that would indicate a sufficient fit. In a similar way, the fit indices of interaction models with a reversed path direction can be compared. The values indicate a weaker fit for the model with the path from PCE to green skepticism, which again may indicate that the path does run from green skepticism to PCE. However, again, the differences are very minimal, so although these results are suggestive, we should avoid drawing too strong a conclusion about this.

## 4. Discussion

An online shopping environment was built to pilot test the conceptual idea that adjusting product information could affect sustainable purchase behavior. It was expected that food products with an explanatory sustainability claim would be purchased more often than products with a sustainability claim only and that the addition of an explanatory health claim to an explanatory sustainability claim would lead to even more sustainable purchases (H1a). Subsequently, PCE (H2a, H2b) and green skepticism (H3a, H3b) were proposed as mechanisms explaining the effects of information type (explanatory vs. claim only) on sustainable purchase behavior. It was also expected that the mediation of green skepticism would be affected by the extent to which consumers hold positive environmental attitudes (H3c).

One of the main findings of this study is that sustainable purchases were not fostered by the addition of an explanatory health claim to an explanatory sustainability claim (H1a), even though several studies highlighted that health attributes of a product are more effective at increasing purchase intentions than sustainability attributes [32,33]. From a social dilemma perspective [15], it was argued that the dilemma of choosing to cooperate for the sake of the collective good versus individual interests could be reduced when consumers are also presented with health attributes because this enforces the individual benefits of buying sustainable products. The findings suggest that a confirmation bias exists that plays a role in affecting people’s refusal to process the information. The lack of significant findings suggests that consumers perceive the costs associated with the collective cooperation (e.g., higher product price of sustainable products) as more important than potential rewards (e.g., environmental benefits, health benefits [34]), although we did not test this in the current study. In line with this, Grunert et al. [35] showed that consumers express concerns about environmental issues, but at the product-related level this concern diminishes and does not influence actual consumption behavior.

As hypothesized in H2b, there was a positive relationship between PCE and sustainable purchase behavior. This is in line with previous studies, although these studies did not investigate PCE in relation to actual purchase behavior [7,16,23,36]. Increasing consumers’ level of PCE through interventions could be an effective strategy to increase their levels of sustainable consumption. However, no evidence was found for H2a, which stated that consumers would have a higher PCE when exposed to explanatory product information. Instead, a negative effect of explanatory product information was found on PCE, meaning that the explanation of a sustainability claim leads to a lower PCE. After exploring this effect in more detail, marginal effects showed that this only occurred for those with low environmental attitudes. One explanation for this finding could be the confirmation bias, which is the human tendency to search for, interpret, favor, and recall information in such a way that it confirms or supports someone’s prior beliefs or values [37]. People unconsciously select and interpret information that supports their existing view and will ignore non-supportive information. In this study, people who already had a low PCE most probably would not consider climate change as something related to human activity or consider it as something that can be reversed and will defend themselves from an inner conflict by avoiding the explanatory information.

In contrast, Vermeir and Verbeke [7] successfully manipulated information in their study to influence PCE, and, in turn, this influenced purchase intentions of an organic dairy product. These authors used a very concrete example of how consumers can indirectly (i.e., with a purchase) exert pressure on a company to ensure better working circumstances for their employees. Differences in information provision could have led to these distinct outcomes, indicating that the formulation of the information could be an important factor that should receive more research attention.

In H3a and H3b, it was expected that explanatory sustainability information would lead to lower skepticism and, in turn, to higher sustainable purchase behavior. The current study did not find support for the effect of green skepticism on sustainable purchase behavior, even though this direct effect has been found in previous studies [31,38]. Instead, an unexpected relationship from green skepticism to PCE was added for model fit, due to the high residual correlation between green skepticism and PCE. To the authors’ knowledge, only one study has investigated this relationship [31], and found a negative effect of green skepticism on PCE. However, this study did not experimentally test this relationship, and the alternative models that were run do not provide conclusive evidence on the direction. Nevertheless, the specification from green skepticism to PCE makes most sense in this study, as green skepticism is operationalized as a feature of the green claims that participants were exposed to (i.e., ‘I do not trust these green claims’). The assessment of the green claim in terms of skepticism was therefore hypothesized to lead to PCE and not vice versa. Future studies could examine whether this relationship is unidirectional, as communication strategies could be adjusted to this (i.e., either focusing on increasing PCE or decreasing green skepticism).

In H3c, it was hypothesized that the effect of an explanatory sustainability claim on green skepticism would be stronger for those with high environmental attitudes compared to low environmental attitudes. The current study revealed that this effect was not only weaker but also became non-significant among those with low environmental attitudes. An explanation could again be linked to the confirmation bias that people have that can lead to cognitive inertia, which is the lack of motivation to generate the distinct cognitive processes needed to attend to a problem or issue that is not in line with someone’s attitudes or mindset at that point; this can even lead to refusing to listen to contradicting information [39].

The reduction in green skepticism among those with high environmental attitudes did not lead to any (indirect) purchase effects. A possible explanation for this finding could be that those with high environmental attitudes already feel like they sufficiently contribute with their purchase behavior and do not see the need to change this. The high mean PCE score of consumers with high environmental attitudes substantiates this explanation (*M* = 5.64, *SD* = 0.87).

The current study has several strengths and limitations that also present opportunities for future research. The first strength is that we used an online shopping environment that was highly identical to an actual online grocery shop that people increasingly use to conduct their grocery shopping, providing the study with high ecological validity. Second, although participants did not actually buy the selected foods, we mimicked the actual consumption behavior as closely as possible, whereas previous research has mainly focused on the attitudes or intentions toward consuming certain food products, thus providing an important insight for the attitude-behavior gap. Third, whereas most studies have focused on the effects of ecolabels providing information about elements related to sustainability, we examined whether a combination of information about sustainability would be more efficient when combined with health-related information.

One of the limitations of the current study is that the experimental stimuli could have played a role in the lack of significant findings between product information and sustainable purchases. Only six product categories were included with a total of 33 products, which could have forced them to choose differently than they normally would when grocery shopping. Second, it is likely that other product characteristics played a larger role in the purchase decision than sustainability, such as favorable brand attitudes or pricing. Third, the average score for information trustworthiness was below the midpoint of the scale in all conditions, which could have affected purchase decisions. Fourth, participants might have been primed by the question order since they started the shopping task shortly after answering questions about their environmental attitudes. This could have led participants to be willing to provide ethically correct answers. Fifth, the current study did not control for the effect of message quantity, though this is potentially an important factor [14]. Lastly, there is a possibility that this study is underpowered, which could be an explanation for the non-significant overall effect (*p* = 0.108) and does not provide very strong evidence that the null hypothesis should be accepted. Another limitation of the sample is the over-representation of females, who are more concerned about the environment than males [40]. Future studies investigating the interplay between sustainability and health communication should employ a larger and more balanced sample size.

Nevertheless, the presence of an online supermarket did guide consumers through a shopping experience, thereby enhancing the ecological validity of this study. Further research should include a more extensive range of product categories, products, and brands in order to more accurately measure consumers’ purchase behavior.

## 5. Conclusions

This study showed that the addition of a health claim to a sustainability claim on food products did not result in more sustainable purchases. Furthermore, an explanatory sustainability claim did not increase sustainable purchase behavior among either consumers with high or low environmental attitudes. Instead, it was revealed that explanatory product information reduces sustainable purchases through PCE, but only for those with low environmental attitudes. Therefore, anticipating changing consumers’ PCE might not be an effective strategy to reach consumers with low environmental attitudes. In other words, the current study shows that adding explanatory sustainability information does more harm than good to consumers’ sustainability purchases. These unexpected results indicate that more research needs to be performed towards informing consumers about sustainability and health attributes in order to increase sustainable purchases and to see whether different framing strategies can steer consumers in a sustainable direction.

## Figures and Tables

**Table 1:** Descriptives of the total sample and divided by condition.

	Total	Sustainability Claim Only	Explanatory Sustainability Claim	Explanatory Sustainability and Health Claim
Sustainable purchase behavior	2.27 (1.46)	2.09 (1.44)	2.69 (1.41)	2.00 (1.48)
PCE	5.42 (1.03)	5.65 (0.95)	5.50 (0.97)	5.09 (1.12)
Green skepticism	3.31 (1.01)	3.53 (0.98)	3.09 (1.03)	3.34 (0.99)
General environmental attitude	5.70 (0.93)	5.68 (0.86)	5.98 (0.80)	5.42 (1.06)
Age	25.57 (9.60)	26.42 (10.24)	22.54 (4.48)	27.94 (12.06)
Gender (% female)	77.2%	84.8%	77.1%	69.7%

**Table 2:** Product categories and products included in the online supermarket.

Bread	Dairy and Sandwich Filling	Pasta	Vegetables	(Vegetarian) Meat	Sauces
White bread *	Filet Americain	Fusilli	Mushrooms	Ground meat (50% beef, 50% pork)	‘Sauce in a bag’ basil *
Whole grain bread *	Grated cheese	Organic—Whole grain penne *	Organic–Mushrooms	Vegetarian ground meat *	Pasta sauce traditional
Brown bread *	Humus tomato-basil *	Whole grain penne	Spinach *	Organic—Ground meat (50% beef, 50% pork)	Organic—Pasta sauce traditional *
Organic—whole-grain bread *	Chicken filet (sliced) *	Fusilli	Zucchini	Chicken filet	Pasta sauce basil
Corn bread *	Vegetarian filet l’americain *		‘Roma’ tomatoes	Vegetarian chicken *	
	Gouda cheese 48+		Organic–tomatoes *		
	Cooked sausage (sliced)		Bell pepper red		
			Organic bell pepper red		

Note: Products including an asterisk (*) were provided with product information.

**Table 3:** Pearson correlations between the continuous variables.

	1.	2.	3.	4.	5.	6.
1. Sustainable purchase behavior	1					
2. PCE	0.33 **	1				
3. Green skepticism	−0.15	−0.27 **	1			
4. Age	−0.23 *	−0.13	−0.20 *	1		
5. Educational level ^a^	0.12	−0.01	0.03	0.58 **	1	
6. Environmental attitude (MS) × experimental condition	0.23 *	0.25 *	0.03	−0.14	0.22 **	1

Note: ^a^ Spearman’s rank correlation coefficient was used for correlations with education level. ** Correlation is significant at the 0.01 level (2-tailed). * Pearson correlation coefficient is significant at the 0.05 level (2-tailed). MS = Median Split.

**Table 4:** Re-specification steps and fit indices.

					RMSEA			
Model	Modifications	*χ*^2^ (df)	*p*	CFI		95%CI LB	95%CI UB	PCLOSE
1_original_	Original model	8.84 (1)	0.003	0.77	0.28	0.13	0.46	0.007
1_final_	1. Addition path PCE—green skepticism 2. Removing paths	4.64 (4)	0.327	0.98	0.04	0.00	0.16	0.451
2_original_	Original model	9.99 (3)	0.019	0.96	0.15	0.06	0.26	0.044
2_addpath_	Addition path PCE— green skepticism	1.15 (2)	0.563	1.00	0.00	0.00	0.17	0.637
2_final_	Removing paths	2.89 (4)	0.546	1.00	0.00	0.00	0.13	0.684

Note: Model 1 represents the model without the interaction term, and Model 2 represents the model with the interaction term. LB = Lower bound. UB = upper bound. The relatively high upper bound of the CI can be attributed to the small sample size.

**Table 5:** Fit indices alternative model.

					RMSEA				
Model	Modifications	*χ*^2^ (df)	*p*	CFI		95%CI LB	95%CI UB	PCLOSE	AIC
Main effects model	With GS → PCE	4.64 (4)	0.327	0.98	0.04	0.00	0.16	0.451	36.64
	With PCE → GS	5.87 (4)	0.209	0.94	0.07	0.00	0.18	0.322	37.87
Interaction model	With GS → PCE	2.89 (4)	0.546	1.00	0.00	0.00	0.13	0.684	48.89
	With PCE → GS	4.07 (4)	0.396	1.00	0.01	0.00	0.15	0.521	50.07
	With GS ←→ PCE	4.07 (4)	0.396	1.00	0.01	0.00	0.15	0.521	50.07

Note: GS = green skepticism. PCE = perceived consumer effectiveness.

## Data Availability

The data presented in this study are available on request from the corresponding author. The data are not publicly available due to the questions being asked in Dutch.

## Appendix A

**Table A1:** List of items per construct.

Construct	Items
Perceived consumer effectiveness	It is worthless for the individual consumer to do anything about pollution
	When I buy products, I try to consider how my use of them will affect the environment and other consumers
	Since one person cannot have any effect upon pollution and natural resource problems, it doesn’t make any difference what I do
	Each consumer’s behavior can have a positive effect on society by purchasing products sold by socially responsible companies
Green skepticism	Most green claims are intended to mislead rather than to inform consumers
	I do not believe the green claims
	Consumers would be better off if the green claims were eliminated from product packaging
	The green claims lead people to believe things that aren’t true
	I feel I have been accurately informed after viewing the green claims
	In general, the green claims present a true picture of the product
	I can trust the green claims
	The green claims do not tell much useful information about products
General environmental attitudes	Our present rate of consumption can be maintained with no ecological problems
	The problems relating to ozone depletion are overstated
	Global warming is not really a problem
	In a crisis, industries will develop solutions to environmental problems
	World population levels are well within what the world can support
	Agricultural productivity will decline in the near future
	Food shortages are possible in the near future, even in developed countries
	Serious shortages of some natural resources will occur in the near future
	Continued use of chemicals in agriculture will damage the environment beyond repair
	Some living things are unnecessarily being threatened with extinction
	Destruction of rainforests will have long term environmental consequences
	Many types of pollution are rising to dangerous levels
